# When the Heart Strikes the Spine: Spinal Cord Infarction Mimicking Longitudinally Extensive Transverse Myelitis (LETM) in Infective Endocarditis

**DOI:** 10.7759/cureus.109860

**Published:** 2026-05-29

**Authors:** Akshaya Kumar, Rajashekar V, Hema K, Mohammed Thaha S, Sahasyaa Adalarasan, TS Santhi, Hariharan C

**Affiliations:** 1 Internal Medicine, Madras Medical College, Chennai, IND; 2 Medicine, Madras Medical College, Chennai, IND

**Keywords:** acute myelopathy, diagnostic challenge, embolic complications, infective endocarditis, letm, longitudinally extensive transverse myelitis, prosthetic valve endocarditis, spinal cord infarction

## Abstract

Infective endocarditis (IE) is a serious infection of the endocardial surface of the heart that is associated with significant morbidity due to systemic embolic complications. Neurological involvement is well recognized but most commonly manifests as ischemic stroke or intracranial hemorrhage due to embolic events. Complete spinal cord infarction as an initial presentation of IE is rare and may mimic acute myelopathy or longitudinally extensive transverse myelitis (LETM), leading to diagnostic challenges. We describe a patient who presented with an acute-onset motor weakness and sensory deficits suggestive of LETM. Initial neuroimaging findings were consistent with LETM. However, further evaluation with blood cultures and echocardiography revealed IE with valvular vegetations, indicating an embolic source and explaining the neurological presentation as spinal cord ischemia secondary to IE. The patient was managed with the timely initiation of appropriate intravenous antimicrobial therapy along with supportive care. This case highlights the importance of considering an underlying embolic etiology in patients presenting with LETM-like acute myelopathy and emphasizes the role of early recognition and timely treatment in improving outcomes and preventing further complications.

## Introduction

Spinal cord infarction is a rare but serious neurological condition caused by interruption of blood supply to the spinal cord, most commonly involving the anterior spinal artery. It accounts for approximately 1%-2% of all strokes and typically presents with a sudden onset of neurological deficits, often resulting in significant morbidity. Common etiologies include aortic pathology, thromboembolism, and, less commonly, embolic events from infective endocarditis (IE) [1].

Longitudinally extensive transverse myelitis (LETM) is defined as a spinal cord lesion extending over three or more contiguous vertebral segments on magnetic resonance imaging (MRI). It is most commonly associated with inflammatory and autoimmune conditions such as neuromyelitis optica spectrum disorders and myelin oligodendrocyte glycoprotein (MOG)-associated disease (MOGAD) [2].

Differentiating spinal cord infarction from LETM can be challenging, as both conditions may demonstrate long-segment spinal cord involvement on MRI and present with acute motor, sensory, and autonomic dysfunction. However, spinal cord infarction typically presents with abrupt onset, non-inflammatory cerebrospinal fluid (CSF) findings, and poor response to immunotherapy, whereas LETM usually has a subacute onset with inflammatory features [3,4].

Among the less common etiologies of spinal cord infarction, IE is an important cause. IE is a microbial infection of the endocardial surface of the heart, most commonly affecting the cardiac valves. It is characterized by vegetations that may fragment and lead to embolization. Neurological complications are common and most often present as ischemic stroke. Spinal cord involvement due to embolic events is rare and may result in spinal cord infarction [5].

In this case, we report a 55-year-old man presenting to the Emergency Outpatient Department (OPD) of Rajiv Gandhi Government General Hospital with acute-onset paraplegia, in whom initial imaging suggested LETM, but further evaluation revealed spinal cord infarction secondary to prosthetic valve IE.

## Case presentation

A 55-year-old man presented with acute-onset low back pain, initially described as a band-like compressive pain just above the groin, non-radiating and not aggravated or relieved by a change in position. This was associated with radiating, shooting pain in the right lower limb, present even at rest and aggravated by walking, coughing, and sneezing, with temporary relief on medication. Over the next two days, he developed progressive weakness associated with reduced sensation below the groin in both lower limbs, resulting in difficulty in using them. This was followed by rapid worsening of weakness over the next two hours, leading to complete inability to move both lower limbs. There was no history of fever, trauma, recent infection, or cranial nerve symptoms.

The patient was a known case of paranoid schizophrenia for the past 15 years and is on irregular treatment with olanzapine and clonazepam. He was also a known case of rheumatic heart disease, for which he had undergone St. Jude mechanical mitral valve replacement, tricuspid valve repair, and left atrial clot removal. He had been on acenocoumarol (Acitrom), which he was taking irregularly. There was no significant family history. There was no history of diabetes, hypertension, or similar complaints in the past. He had a history of smoking 2-3 cigarettes per day, which he stopped two years ago. He was not an alcoholic.

On examination, the patient was conscious, oriented, and afebrile, with a recorded blood pressure of 110/60 mmHg. Neurological examination showed hypotonia and bilaterally symmetrical weakness in both lower limbs, with power 0/5. Deep tendon reflexes in both lower limbs were absent. Sensory examination showed decreased sensation below the T10 level. Autonomic examination showed urinary retention, constipation, impaired anal reflex, and decreased anal tone. Systemic examination was normal. Clinical examination findings are summarized in Table 1.

**Table 1:** Summary of clinical examination findings.

Examination	Findings
Consciousness and orientation	Conscious and oriented
Higher mental functions	Memory, speech, and language were intact
Cranial nerves	Normal
Tone	Hypotonia in both lower limbs
Motor power, lower limbs	0/5 bilaterally
Motor power, upper limbs	5/5
Deep tendon reflexes	Absent in both lower limbs
Superficial reflexes	Absent, including abdominal and cremasteric reflexes
Bulbocavernosus reflex	Absent
Plantar response	Extensor bilaterally
Sensory examination	Decreased touch, pain, vibration, and temperature sensation below T10
Autonomic involvement	Urinary retention, constipation
Anal reflex and tone	Impaired reflex and decreased tone
Cerebellar signs	Absent
Extrapyramidal signs	Absent
Meningeal signs	Absent
Kernig’s sign	Negative
Brudzinski’s sign	Negative
Spine examination	Mild localized tenderness over lumbar vertebrae
Straight leg raising test	Negative bilaterally
Cardiovascular examination	S1 and S2 were present with a prosthetic valve click. The rhythm was irregular, and there was no murmur
Respiratory system examination	Respiratory system examination showed bilateral air entry with normal vesicular breath sounds
Abdominal examination	The abdomen was soft, with normal bowel sounds present and no organomegaly

Baseline laboratory and infectious workup findings are summarized in Table 2. MRI of the dorsal spine with contrast revealed intramedullary T2 hyperintensity involving the lower thoracic spinal cord segments from D9 to D12 extending to the conus medullaris, with mild diffusion restriction and contrast enhancement (Figures 1, 2). The lesion involved both the anterior and posterior segments of the spinal cord, suggesting extensive cord involvement (Figure 3). These findings were suggestive of a LETM. As the acute onset and MRI findings were consistent with LETM, and in the absence of fever or clinical evidence of infection, the patient was initially treated with high-dose intravenous methylprednisolone (1 g once daily for five days). However, no significant neurological improvement was observed following steroid therapy.

**Table 2:** Baseline laboratory and infectious workup.

Test result	Patient value	Normal value/reference range	Interpretation
Red blood cell count	5.1 million cells/µL	Male: 4.6-6.2 million cells/µL; female: 4.2-5.4 million cells/µL	Within normal limits
Hemoglobin	14.2 g/dL	Male: 13-18 g/dL; female: 12-16 g/dL	Within normal limits
Hematocrit	43%	Male: 40%-55%; female: 36%-48%	Within normal limits
Total leukocyte count	7,800 cells/µL	4,500-11,000 cells/µL	Within normal limits
Platelet count	2.6 lakh/µL	1.5-4.0 lakh/µL	Within normal limits
Mean corpuscular volume	90 fL	80-100 fL	Normocytic red cells
Mean corpuscular hemoglobin	29 pg/cell	27-32 pg/cell	Within normal limits
Mean corpuscular hemoglobin concentration	34 g/dL	32-36 g/dL	Within normal limits
Blood urea	28 mg/dL	15-40 mg/dL	Within normal limits
Serum creatinine	0.9 mg/dL	0.6-1.3 mg/dL	Normal renal function
Aspartate aminotransferase	22 U/L	8-33 U/L	Within normal limits
Alanine aminotransferase	25 U/L	4-36 U/L	Within normal limits
Alkaline phosphatase	86 U/L	20-130 U/L	Within normal limits
Total bilirubin	0.8 mg/dL	0.1-1.2 mg/dL	Within normal limits
Serum albumin	4.2 g/dL	3.4-5.4 g/dL	Within normal limits
Serum sodium	139 mEq/L	135-145 mEq/L	No electrolyte imbalance
Serum potassium	4.2 mEq/L	3.5-5.0 mEq/L	No electrolyte imbalance
Serum chloride	101 mEq/L	96-106 mEq/L	No electrolyte imbalance
International normalized ratio	3.04	0.8-1.1; therapeutic range on anticoagulation usually 2.0-3.0	Elevated, consistent with anticoagulation therapy

**Figure 1:**
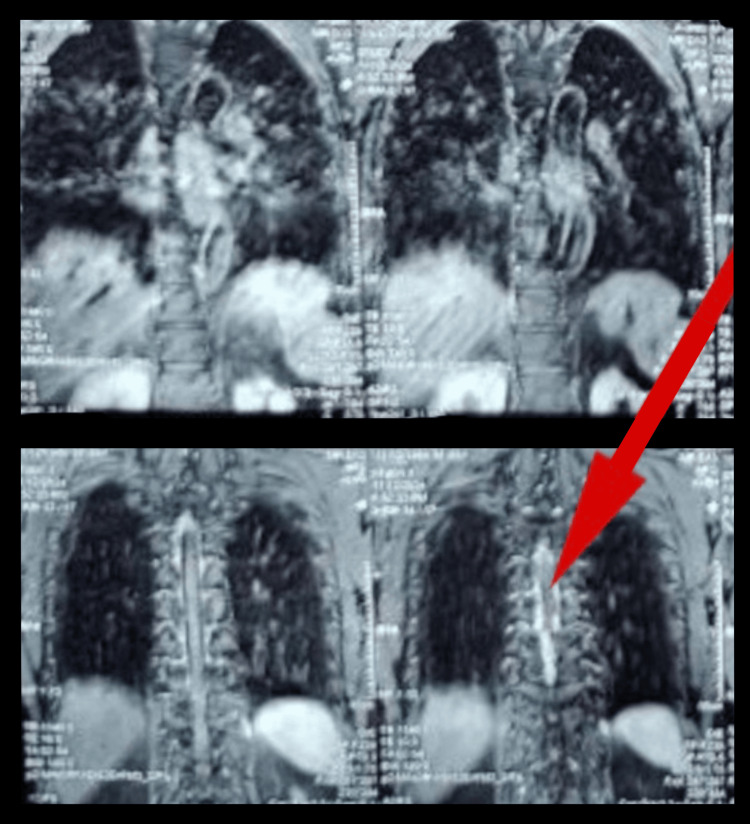
Multiple axial sections of the dorsal spine with whole spine screening demonstrating long-segment intramedullary hyperintensity, consistent with longitudinally extensive transverse myelitis (LETM). Long-segment intramedullary T2 hyperintensity, initially interpreted as consistent with LETM. LETM was the initial radiological diagnosis; however, subsequent clinical, microbiological, and echocardiographic findings supported a diagnosis of presumed embolic spinal cord infarction secondary to infective endocarditis.

**Figure 2:**
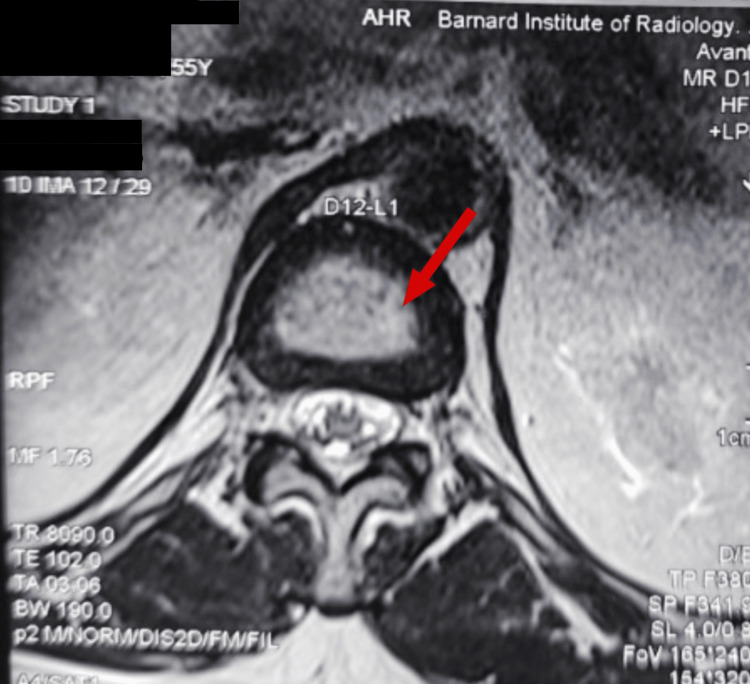
Sagittal magnetic resonance imaging (MRI) of the dorsal spine showing continuous T2 hyperintensity extending from D9 to D12 with involvement of the conus medullaris.

**Figure 3:**
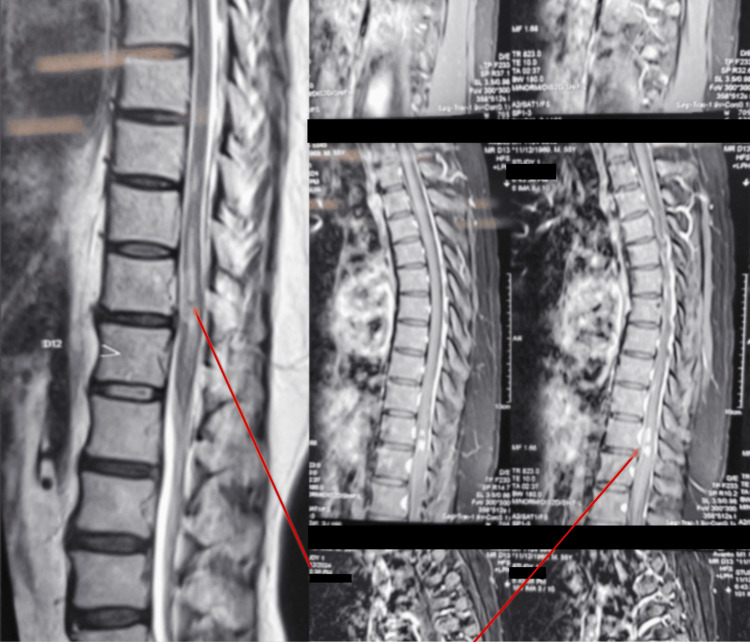
Magnetic resonance imaging (MRI) demonstrates intramedullary T2 hyperintensity involving both the anterior and posterior segments of the spinal cord, suggesting extensive cord involvement. The red arrows indicate intramedullary T2 hyperintensity, suggesting extensive spinal cord involvement and longitudinally extensive transverse myelitis (LETM). However, subsequent clinical, microbiological, and echocardiographic findings supported a diagnosis of presumed embolic spinal cord infarction secondary to infective endocarditis.

CSF analysis was performed, which demonstrated elevated protein levels (110 mg/dL) with normal glucose and an acellular picture. CSF viral panel and cultures were negative (Table 3). These findings did not support an infective etiology. Autoimmune evaluation including serum aquaporin-4 (AQP4) and MOG antibodies was negative. Visual evoked potential was performed and found to be normal. Nerve conduction study of all four limbs was normal.

**Table 3:** Cerebrospinal fluid (CSF) analysis. CSF glucose was 79 mg/dL (normal, with corresponding serum glucose of 121 mg/dL; CSF glucose is typically approximately 60%-70% of the serum glucose concentration). LDH: lactate dehydrogenase; CMV: cytomegalovirus; HSV: herpes simplex virus; EBV: Epstein-Barr virus

Parameter	Value	Interpretation	Reference range
Protein	110 mg/dL	Elevated	15-45 mg/dL
Glucose	79 mg/dL	Normal	45-80 mg/dL
Cell count	Acellular	No pleocytosis	0-5 cells/mm³
LDH	32 IU/L	Within normal limits	<40 IU/L
Viral panel (CMV, HSV, EBV)	Negative	No viral infection	Negative
CSF culture	No growth	No bacterial infection	Sterile
Fungal studies/India ink	Negative	No fungal infection	Negative

MRI of the brain with magnetic angiography and magnetic venography showed a few foci of blooming in the bilateral parietal and right occipital regions, suggestive of microhemorrhages, with no evidence of acute infarction or major intracranial pathology (Figure 4).

**Figure 4:**
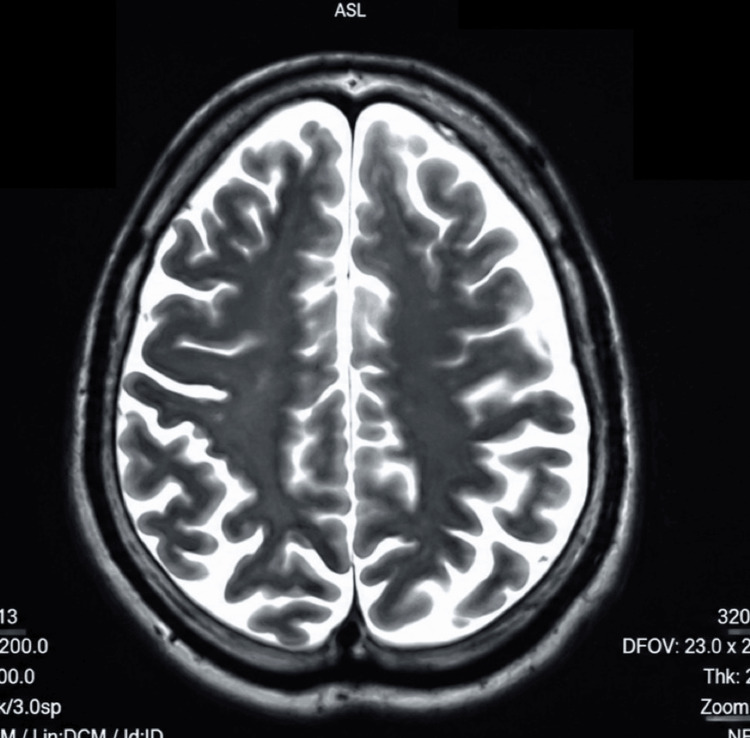
Magnetic resonance imaging (MRI) brain showing no acute focal infarct or demyelinating lesions, with incidental findings of mild generalized cerebral atrophy and microhemorrhages.

During the hospital course, the patient developed a fever, prompting a blood culture evaluation. Blood cultures grew *Staphylococcus aureus*, which was considered the primary causative organism for IE. Subsequent cultures yielded *Klebsiella pneumoniae*, suggesting polymicrobial bacteremia. Given the presence of a prosthetic mitral valve and persistent bloodstream infection, IE was strongly suspected. Transthoracic echocardiography (TTE) did not demonstrate vegetations; however, transesophageal echocardiography (TEE) revealed a vegetation measuring approximately 10 x 3 mm on the prosthetic mitral valve (Figure 5). According to the Modified Duke Criteria, the patient fulfilled two major criteria - positive blood cultures with a microorganism consistent with IE and echocardiographic evidence of endocardial involvement in the form of prosthetic valve vegetation - establishing a diagnosis of definite IE. The patient also fulfilled minor criteria, including a predisposing prosthetic mitral valve, documented fever, and a vascular phenomenon in the form of presumed embolic spinal cord ischemia.

**Figure 5:**
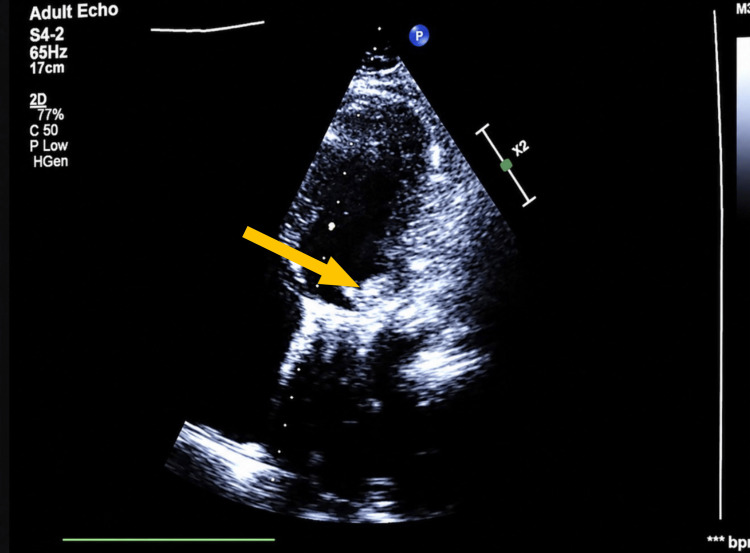
Transesophageal echocardiography showing prosthetic mitral valve vegetation.

In view of the polymicrobial bacteremia and the susceptibility profile of the isolated organisms, antimicrobial therapy was individualized rather than standard guideline-directed therapy. *S. aureus* was sensitive to cefoxitin, linezolid, tetracycline, and trimethoprim-sulfamethoxazole, while *K. pneumoniae* was sensitive to amikacin, piperacillin-tazobactam, cefotaxime, meropenem, and trimethoprim-sulfamethoxazole. Accordingly, the patient received oral linezolid 600 mg twice daily and trimethoprim-sulfamethoxazole 480 mg twice daily for a total duration of seven weeks. Repeat TEE showed a decrease in the size of the vegetation, and repeat blood cultures were negative. There was no significant neurological improvement after two months of illness. The patient had persistent bilateral lower limb weakness (power 1/5), with mild improvement in bowel sensation. The patient was continued on anticoagulation and discharged with a urinary catheter in situ, with advice for follow-up and rehabilitation. The chronological timeline of the patient’s clinical presentation, diagnostic evaluation, treatment, and outcome is summarized in Table 4.

**Table 4:** Timeline of clinical presentation, diagnostic evaluation, treatment, and outcome. MRI: magnetic resonance imaging

Timeline	Clinical events
Presentation	Acute-onset bilateral lower limb weakness with sensory and bladder involvement. MRI spine showed a long-segment intramedullary lesion extending from D9-D12 to the conus medullaris, initially interpreted as longitudinally extensive transverse myelitis (LETM).
Initial management	In the absence of fever or overt clinical evidence of infection, the patient was treated with intravenous methylprednisolone for presumed inflammatory myelopathy.
Subsequent clinical course	The patient developed a fever during hospitalization, prompting further evaluation for an infectious etiology.
Microbiological evaluation	Blood cultures grew *Staphylococcus aureus*, which was considered the causative organism for infective endocarditis. Subsequent serial blood cultures yielded *Klebsiella pneumoniae*, suggesting polymicrobial bacteremia.
Cardiac evaluation	Transthoracic echocardiography (TTE) did not demonstrate vegetations. Given persistent clinical suspicion, transesophageal echocardiography (TEE) was performed and revealed a mobile vegetation measuring approximately 10 x 3 mm on the prosthetic mitral valve.
Definitive diagnosis	Based on positive blood cultures and echocardiographic evidence of vegetation, the patient fulfilled the Modified Duke Criteria for definite prosthetic valve infective endocarditis. The spinal cord lesion was subsequently considered most consistent with a presumed embolic spinal cord infarction presenting as an LETM-like syndrome.
Treatment	In view of the polymicrobial bacteremia and susceptibility profile of the isolated organisms, antimicrobial therapy was individualized. The patient received linezolid 600 mg twice daily and trimethoprim-sulfamethoxazole 480 mg twice daily for seven weeks.
Follow-up	Repeat blood cultures became sterile, and follow-up TEE demonstrated a reduction in vegetation size, indicating microbiological and echocardiographic response to treatment.
Outcome	At two months after symptom onset, there was no significant motor recovery (lower limb power 1/5 bilaterally), although mild improvement in bowel sensation was noted. The patient was discharged on anticoagulation with an indwelling urinary catheter and advised regular follow-up.

Although the initial MRI appearance was suggestive of LETM, several clinical and laboratory findings argued against an inflammatory myelopathy. The neurological deficits developed acutely rather than showing the more gradual progression typically associated with LETM. CSF analysis was acellular, viral studies were negative, and an extensive autoimmune workup, including relevant autoantibody testing, did not identify an inflammatory or demyelinating etiology. While the imaging pattern was not restricted to a single spinal arterial territory, embolic spinal cord ischemia can produce atypical and extensive lesions involving multiple vascular distributions. Furthermore, the patient demonstrated less response to high-dose intravenous corticosteroid therapy. Subsequent evaluation established definite prosthetic valve IE based on the Modified Duke Criteria, providing a plausible source for embolic vascular injury. Following antimicrobial therapy and anticoagulation, blood cultures became sterile, repeat echocardiography demonstrated a reduction in vegetation size, and motor power improved to 1/5 in both lower limbs. Considering the acute onset of symptoms, negative CSF and autoimmune investigations, less response to corticosteroids, the presence of definite IE, and partial neurological improvement following treatment directed at the underlying infectious and vascular process, the spinal cord lesion was considered most consistent with a presumed embolic spinal cord infarction presenting as an LETM-like syndrome rather than true LETM.

## Discussion

IE is a serious infection of the endocardial surface of the heart and remains associated with significant morbidity due to systemic complications and embolic events. The 2023 European Society of Cardiology (ESC) guidelines identify embolization as a major complication of IE. Large clinical studies report neurological involvement in approximately 20%-40% of patients, most commonly presenting as ischemic stroke or intracranial hemorrhage [5,6].

The neurological manifestations in IE are primarily due to embolization from infected valvular vegetations composed of fibrin, platelets, and microorganisms, which can fragment and travel through the bloodstream, leading to arterial occlusion. While cerebral embolism is the most common presentation, the spinal cord may also be affected when its vascular supply is compromised, particularly involving the anterior spinal artery, resulting in sudden-onset motor weakness, sensory deficits, and autonomic dysfunction. Clinically, this may mimic acute transverse myelopathy, including LETM, especially when extensive segmental involvement is seen on imaging. Early MRI findings may be normal or inconclusive, further adding to diagnostic difficulty [7].

Spinal cord infarction secondary to IE has been reported only in a few cases in the literature. A 1993 study described polymicrobial IE presenting with anterior spinal artery syndrome and quadriplegia [8]. A report in 2017 documented aortic valve endocarditis with spinal cord infarction associated with concurrent cerebral embolic infarcts [9]. Another case in 2018 reported *Streptococcus mitis* endocarditis presenting with focal lower limb weakness due to thoracic cord infarction in a patient with underlying malignancy [10]. A more recent report in 2021 described rapidly progressive IE with widespread septic embolization involving multiple organs, including the spinal cord [11].

Pawar et al. reported a patient who was initially diagnosed with acute transverse myelitis based on clinical and MRI findings; however, repeat MRI subsequently demonstrated spinal cord and vertebral body infarction, emphasizing the importance of serial imaging in differentiating spinal cord infarction from inflammatory myelopathies [12]. Ibrahim et al. described a classical case of anterior spinal cord infarction presenting with sudden-onset paraplegia and preserved dorsal column sensations, with MRI findings consistent with ischemia in the anterior spinal artery territory [13]. Novy reviewed the clinical spectrum of spinal cord infarction and highlighted that, although anterior spinal artery syndrome is the most common presentation, posterior, unilateral, central, and transverse infarctions may also occur depending on the vascular territory involved [14]. Similar to the case reported by Pawar et al., our patient was initially considered to have LETM based on clinical and radiological findings. However, the acute onset of symptoms, acellular CSF, negative viral studies, negative autoimmune evaluation, less response to corticosteroid therapy, and the presence of definite IE favored a diagnosis of presumed spinal cord infarction. In addition, unlike the classical anterior spinal artery territory involvement described by Ibrahim et al., our patient demonstrated extensive spinal cord involvement affecting both anterior and posterior cord regions on MRI. This observation is consistent with the broader spectrum of spinal cord infarction patterns described by Novy and supports the possibility of a more extensive vascular insult in the setting of IE.

In our case, the patient presented with features of acute myelopathy that were clinically suggestive of LETM, which was later considered most consistent with presumed spinal cord infarction secondary to embolic phenomena from IE. The absence of early definitive cardiac features contributed to the diagnostic difficulty. This highlights the importance of considering an underlying embolic etiology in patients presenting with LETM-like acute myelopathy, particularly when clinical features suggest a systemic infectious source.

Taken together, this case emphasizes the importance of early suspicion and evaluation for IE in cases of unexplained acute myelopathy. Early blood cultures, echocardiographic assessment, and timely initiation of appropriate antimicrobial therapy are essential to prevent further embolic events and improve clinical outcomes [5,7]. This case demonstrates presumed spinal cord infarction secondary to IE presenting as an LETM-like acute myelopathy with extensive spinal cord involvement. It highlights the diagnostic challenge of distinguishing spinal cord infarction from inflammatory myelopathies and emphasizes the importance of evaluating for an embolic source in patients presenting with acute LETM-like syndrome.

The limitation of this study is the absence of dedicated vascular imaging to directly demonstrate spinal cord ischemia. In addition, histopathological confirmation was not feasible.

## Conclusions

IE should be considered in the differential diagnosis of acute myelopathy, particularly when the clinical presentation is abrupt and accompanied by features suggestive of a systemic infectious process. Spinal cord ischemia secondary to embolic phenomena can mimic LETM on imaging, creating a diagnostic challenge. Early recognition, prompt blood cultures, and echocardiographic evaluation are essential for identifying an underlying embolic source and initiating appropriate therapy.

In this case, we report a patient with prosthetic valve IE who presented with an LETM-like acute myelopathy and was subsequently considered to have presumed spinal cord infarction secondary to embolic phenomena. The diagnosis was supported by the acute onset of neurological deficits, acellular CSF, negative viral and autoimmune workup, less response to corticosteroid therapy, positive blood cultures, and echocardiographic evidence of prosthetic valve vegetation fulfilling the Modified Duke Criteria for definite IE. Following antimicrobial therapy and anticoagulation, blood cultures became sterile, repeat echocardiography demonstrated a reduction in vegetation size, and the patient showed partial neurological improvement with lower limb motor power improving to 1/5 bilaterally. This case highlights the importance of considering an embolic etiology in patients presenting with acute LETM-like syndromes, particularly when conventional inflammatory investigations are unrevealing.
